# Beware of risk for increased false positive rates in genome-wide association studies for phenotypic variability

**DOI:** 10.3389/fgene.2013.00093

**Published:** 2013-05-21

**Authors:** Xia Shen, Örjan Carlborg

**Affiliations:** ^1^Division of Computational Genetics, Department of Clinical Sciences, Swedish University of Agricultural SciencesUppsala, Sweden; ^2^School of Technology and Business Studies/Statistics, Dalarna UniversityBorlänge, Sweden

Performing genome-wide association studies (GWAS) to identify genes regulating the between-genotype variability, rather than the mean, is a new promising approach for dissecting the genetics of complex traits. Using this strategy, Yang et al. (2012) successfully identified and replicated the FTO locus and showed that it has a role in regulating the between-genotype variance heterogeneity of human body mass index using a parametric regression model. This finding illustrates the potential clinical contribution of this type of inheritance and that it is not only a feature of model organisms (e.g., Queitsch et al., 2002; Sangster et al., 2008; Gangaraju et al., 2011; Jimenez-Gomez et al., 2011; Christine et al., 2012; Shen et al., 2012). As it is likely that this paper will increase the interest for applying this methodology in other human and experimental populations, we think that it is important to make prospective users aware that one need to be careful when applying similar methodology to smaller datasets than those used by Yang et al.

Yang et al. (2012) noticed that the mapping of variance-controlling loci is prone to inflated test statistics when the minor allele frequency (MAF) is small, but provided no further explanation for this. Here, we will briefly explain why such observation is only half true and why GWAS analyses to detect variance heterogeneity is inherently sensitive to unbalanced data, and why researchers aiming to perform similar analyses need to be careful to avoid reporting false positive signals.

The basis for the sensitivity of variance-heterogeneity GWAS analyses is that the commonly applied statistical tests for variance heterogeneity, including e.g., regression using the squared Z-score, the Levene test (Levene, 1960) and the Brown–Forsythe test (Brown and Forsythe, 1974), are biased when applied to imbalanced samples. The major reason for this is that the distribution of the variance often deviates from normality as it: (1) is bounded at zero; (2) has a distribution skewed to the right; (3) has a variance depending on its mean. Such deviations leads to violations of, e.g., the Gauss–Markov assumptions in a regression model (Plackett, 1950), which could cause problems such as those highlighted here. This bias is usually not discussed in the standard statistics literature as it appears only when the samples are severely imbalanced and is not sufficiently strong to be of importance when the tests are used in situations without excessive multiple-testing. GWAS analyses, however, goes well beyond normal statistical theory by doing hundreds of thousands to millions of tests in severely imbalanced samples. As we will show below, these situations could lead to problems with type I errors, even when stringent Bonferroni-corrected thresholds are used, unless caution is taken in the design of the study and in the quality control of the results.

**Figure 1:**
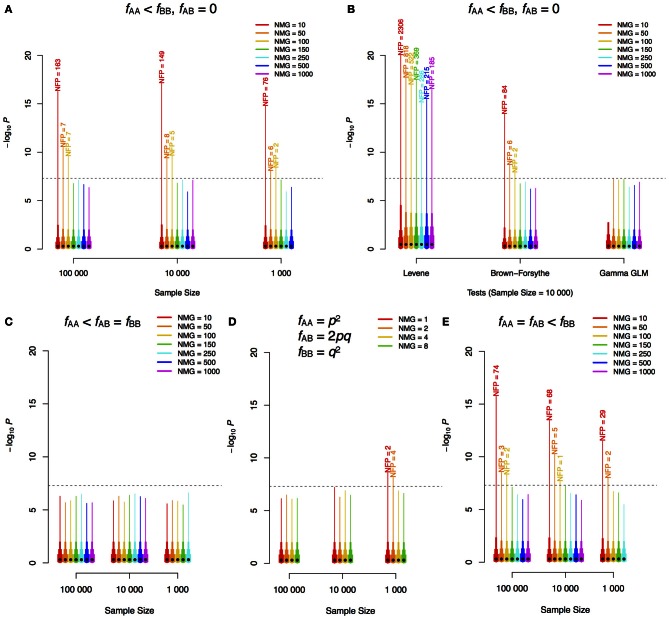
**–log_10_*P*-value distribution for different scenarios of GWAS for phenotypic variability.** Different sample sizes (*n*) and numbers of individuals in the minor genotype class (NMG = *nf*_AA_) were simulated. 1,000,000 replicates for each combination of *n* and NMG were performed with phenotypes simulated as white noise from a standard normal distribution (i.e., no genetic effects). The same method as that employed by (Yang et al., 2012), regression using the squared Z-scores, was used in the analyses of **(A,C–E)**. Two genotype classes were simulated in **(A)** and **(B)**, and three were simulated in **(C)** (one minor class), **(D)** (Hardy–Weinberg equilibrium) and **(E)** (two minor classes). The dashed horizontal lines show the Bonferroni corrected threshold for 1,000,000 tests. The black dot on each bar indicates the median of the 1,000,000 scores, and the top ends of the bars with different widths indicate 85, 95, 99, and 100% (maximum) quantiles of the scores. The labels on top of the bars are the corresponding numbers of false positives (NFP) above the threshold. *f*, frequency; *p*, minor allele frequency; *q*, 1–*p*; GLM, generalized linear model.

To illustrate this inherent problem in the statistical methodology used to test for variance heterogeneity, we used simple simulations in two populations: one with two genotypes: AA and BB and one with three genotypes: AA, AB, and BB. In the simulations, the number of individuals in the minor genotype class (NMG) was varied in populations of increasing sizes. Phenotypes were simulated as pure noise from a standard normal distribution, i.e., all significant signals are false-positives as no genetic effect was simulated. We performed 1,000,000 tests for a variance difference for each combination of population-size and NMG. The number of tests that exceeded the Bonferroni-corrected significance threshold for 1,000,000 independent tests was counted to provide an estimate of the expected number of false positive signals in a genome-scan. As shown in Figure 1A, when there are only two genotype classes, the type I error rate can be very large if the NMG contains fewer than 100 observations when using regression on the squared Z-score, and this cannot be overcome by increasing the total sample-size. The Levene and Brown–Forsythe tests also show such an inflation of false positives (Figure 1B), but use of a Gamma regression model, which accounts for the fact that the squared Z-score follows a chi-square distribution, overcomes this problem. Populations with three genotypes will, in practice, be more robust when the allele substitution model implemented in most GWAS-software is used (i.e., when regression on all three genotypes is used to estimate the additive effect). Inflated type I error rates are then observed only when the intermediate-size genotype class (i.e., in practice most often the heterozygotes) contains fewer than 100 individuals (Figures 1C–E). It should be noted, however, that if the additive genetic effect is estimated as a contrast between the homozygotes (ignoring heterozygotes) or if the dominance effect is included in the model, the bias will be determined by NMG in the same way as when only two genotype classes are present in the population. In our simulations, false signals appear only when the number of observations is lower in the high-variance class (not shown). When the low-variance class has fewer observations, the test is underpowered, which is a likely reason for the lack of false positives. This asymmetry in power has earlier been discussed by Shen et al. (2012).

In practice this means that researchers aiming to perform a GWAS for detection of genes affecting the between-genotype variance difference need to be aware that they may take a considerable risk of obtaining excessive numbers of false positives when the allele-frequencies differ and the NMG is associated with the high-variance estimate. This applies even when stringent multiple-testing corrections are used. We therefore advise that results should be interpreted with caution when (i) the genetic effect in the model is a contrast between two genotype classes and there are *less than 100 observations in the minor genotype class*, or (ii) the genetic effect in the model is estimated using observations from three genotype classes and there are *less than 100 observations in the intermediate-size genotype class*. In such situations, a Gamma generalized linear models (GLM) should be applied to further examine the results.
